# Methodological Advancements for Investigating Intra-tumoral Heterogeneity in Breast Cancer at the Bench and Bedside

**DOI:** 10.1007/s10911-020-09470-3

**Published:** 2020-12-09

**Authors:** Mokryun Baek, Jeffrey T. Chang, Gloria V. Echeverria

**Affiliations:** 1 Lester and Sue Smith Breast Center, Baylor College of Medicine, Houston, TX 77030 USA; 2 Department of Medicine, Baylor College of Medicine, Houston, TX 77030 USA; 3 Department of Pharmacology and Integrative Biology, University of Texas Health Science Center at Houston, Houston, TX 77030 USA; 4 Dan L. Duncan Cancer Center, Baylor College of Medicine, Houston, TX 77030 USA; 5 Department of Molecular and Cellular Biology, Baylor College of Medicine, Houston, TX USA

## Abstract

There is a major need to overcome therapeutic resistance and metastasis that eventually arises in many breast cancer patients. Therapy resistant and metastatic tumors are increasingly recognized to possess intra-tumoral heterogeneity (ITH), a diversity of cells within an individual tumor. First hypothesized in the 1970s, the possibility that this complex ITH may endow tumors with adaptability and evolvability to metastasize and evade therapies is now supported by multiple lines of evidence. Our understanding of ITH has been driven by recent methodological advances including next-generation sequencing, computational modeling, lineage tracing, single-cell technologies, and multiplexed *in situ* approaches. These have been applied across a range of specimens, including patient tumor biopsies, liquid biopsies, cultured cell lines, and mouse models. In this review, we discuss these approaches and how they have deepened our understanding of the mechanistic origins of ITH amongst tumor cells, including stem cell-like differentiation hierarchies and Darwinian evolution, and the functional role for ITH in breast cancer progression. While ITH presents a challenge for combating tumor evolution, in-depth analyses of ITH in clinical biopsies and laboratory models hold promise to elucidate therapeutic strategies that should ultimately improve outcomes for breast cancer patients.

## Introduction

Breast cancer is the most frequently diagnosed cancer and the leading cause of cancer deaths in women worldwide [1]. In addition to the *inter-patient heterogeneity*, across patients, of protein levels of established prognostic and predictive biomarkers (estrogen receptor, ER, progesterone receptor, PR, and human epidermal growth factor receptor 2, HER2) [2], the cells *within* each tumor are also diverse with respect to their somatic mutations, gene expression and epigenetic profiles, and proteomic and metabolic programming. This intra-tumor heterogeneity (ITH) is a major obstacle for diagnosis, prognostic prediction, and standardization of treatment for breast cancers. ITH can be appreciated in different areas within a tumor (spatial heterogeneity) at a single point in time, temporally throughout tumor progression, or amongst primary and metastatic lesions within an individual patient. Thus, conventional sequencing from a single biopsy at a single time point provides only a partial view of the full molecular complexity of a patient’s tumor(s) [3].

ITH can originate through a variety of mechanisms involving cancer stem-like cell (CSC) hierarchies [4] and clonal evolution of somatic genomic aberrations [5]. Both concepts postulate that tumors originate from a single tumor-initiating cell (TIC) that has acquired multiple molecular alterations and developed indefinite proliferative potential. In the CSC model, cancers are hierarchically organized with a stem cell-like population, initiating tumor growth through self-renewal and differentiation [6]. In the clonal evolution model, genomic instability results in accumulation of somatic mutations and/or copy number alterations (CNAs) during disease progression or when faced with selective pressures. These processes are not mutually exclusive. In fact, they are potentially complementary, creating a complex tumor ecosystem with multiple layers of heterogeneity. Furthermore, heterogeneity of nutrient availability and interaction with stromal populations can exist within tumors [7]. The important roles of stromal populations in breast cancer have been recently reviewed [8, 9].

ITH within tumor cells exists at the genomic, transcriptomic, epigenetic, proteomic, and functional levels. This complex ITH is now understood to have crucial implications for breast tumor biology based on several observations: (1) some patterns of ITH have been found to be reproducibly altered in metastatic or therapy-resistant tumor biopsies, (2) levels of ITH have been found to correlate with clinical outcomes in breast cancer patients, and (3) direct experimental evidence in laboratory models has established a functional role for inter-clonal cooperativity in some contexts. Numerous methods have been used to identify ITH in breast cancer specimens, including bulk and single-cell genomic and transcriptomic sequencing, lineage tracing, analysis of circulating tumor cells and DNA, and *in situ* analyses on tissues. These methods have been implemented in patient tumor biopsies and laboratory models including cell lines, genetically engineered mouse models (GEMMs), and patient-derived xenograft (PDX) mouse models. Taken together, this diversity of approaches and models is painting an ever-clearer picture of the heterogeneous composition of tumors, which is providing a deeper understanding of breast cancer biology.

## Bulk Methods to Assess ITH

### Analysis of Tumor Biopsies From the Clinic

Breast cancers fall into three major subtypes based on expression of clinically relevant markers: ER/PR+, HER2+, and triple negative (TNBC). Multi-platform genomic, transcriptomic, epigenomic, and proteomic profiling have elucidated molecular subtypes of breast cancer [10–13]. In particular, PAM50 subtyping, a microarray-based 50-gene signature classifying breast cancers into five intrinsic subtypes (Luminal A, Luminal B, HER2-enriched, Basal-like, and normal-like), provides prognostic and predictive classification of breast tumors [11, 13]. The development of high-depth genomic sequencing technologies has enabled computational estimation of genomic ITH, or subclonal architecture, within tumors in each of these subtypes. Genomic sequencing and immuno-fluorescence *in situ* hybridization (iFISH) using chromosomal probes in small patient cohorts have provided contrasting evidence as to whether the degree of genomic ITH in primary untreated tumors correlates with breast cancer subtype [14, 15]. This point will need to be more thoroughly vetted through genome-wide analyses of larger patient cohorts spanning the major subtypes. Here we discuss studies of clinical biopsies, thus providing a portrait of ITH in biopsies obtained directly from breast cancer patients (Table 1).

**Table 1:** Models and methods for investigating ITH in breast cancer

		Sample / model				Methodology		
Setting	Sample	Pros	Cons	Method type	ITH analyzed	Pros	Cons	Refs.
Clinical	Tumor biopsies	• unmanipulated human biopsies • spatial sampling is possible	• difficulty in separating tumor and stroma • difficulty in obtaining numerous biopsies especially from rare breast cancer subtypes • difficult to obtain longitudinal samples at meaningful time points • biological replicates impossible • environment and genetic backgrounds are diverse • quality of sample is variable	Bulk profiling	• Genomic (DNA)	• relatively low cost • numerous computational methods available	• computational methods are estimations of true genomic architecture • the majority of genomic aberrations are passengers	[14–32]
					• on-tissue (DNA, RNA, protein)	• spatial ITH discernable • correlations with morphological features • broad availability of clinical FFPE specimens	• limit of detection for low-abundance DNA or RNA targets • limited by availability of antibodies for proteins of interest	[14, 33]
				Single-cell profiling	• Genomic (DNA) • transcriptomic (RNA)	• high resolution to more accurately model clonal architecture • ability to separate subpopulations based on profiles	• high cost • difficulty in obtaining sufficient cell numbers and high-quality cell samples	[12, 14, 23, 34–48]
	Liquid biopsies	• minimally invasive • ease of longitudinal biopsies • can encompass ITH from multiple disease sites	• may not capture all tumor cell subpopulations • small cell numbers obtainable • biological replicates impossible	Bulk profiling	• Genomic (DNA)	same as above	same as above	[49–52]
				Single-cell profiling	• Genomic (DNA) • transcriptomic (RNA)	same as above	same as above	[53–55]
Preclinical	GEMM	• possible to obtain biological replicates • fully intact immune system • native tumor-stroma interactions • controllable genetic manipulations/ reporters/ labeling • injectable models of metastasis	• non-human tumor cells • harbor less genomic complexity than human tumors • rapid timescale of tumorigenesis relative to human cancer • low rate of spontaneous metastasis • high cost and effort of mouse breeding • models for some breast cancer subtypes lacking	Bulk profiling	• Genomic (DNA)	same as above	cannot computationally eliminate stroma	[56]
				Single-cell profiling	• Transcriptomic (RNA)	same as above	cannot computationally eliminate stroma	[57, 58]
				Functional experiments	• CSC properties • inter-clonal cooperation • lineage tracing • intravital/ tissue imaging	• functional relevance beyond observational studies • dynamics observable through longitudinal studies • spatial ITH discernable on tissue • intravital imaging of *in vivo* cell behavior	• lengthy experimentation timelines • high cost of lentiviral libraries/sequencing/imaging	[59–69]
	PDX	• possible to obtain biological replicates • human biopsy tumor cells • retain molecular complexity of human tumors • inter-patient heterogeneity represented among models • platform for preclinical trials • injectable models of metastasis	• lack of intact immune system • cross-species tumor-stroma interactions • selection of subclones can occur • high cost of immune-compromised mouse purchase and maintenance	Bulk profiling	• Genomic (DNA)	• ease of computationally discerning tumor from stroma	same as above	[24, 25, 70–74]
				Single-cell profiling	• Genomic (DNA)	• ease of computationally discerning tumor from stroma	same as above	[75, 76]
				Functional experiments	• CSC properties • lineage tracing • intravital/tissue imaging	same as above	• difficulty in generating long-term *ex vivo* cultures	[4, 77–79] [24, 66, 73, 80–83]
	Cell lines	• use of biological replicates • low cost • ease of genetic and functional manipulation • availability of injection models for xenograft studies	• lack of the full repertoire of ITH • *in vitro*-specific selection can occur • lack of microenvironment	Bulk profiling	• Genomic (DNA)	same as above	same as above	[84]
				Single-cell profiling	• Genomic (DNA) • transcriptomic (RNA) • epigenetic (chromatin)	same as above	same as above	[84, 85–89]
				Functional experiments	• CSC properties • inter-clonal cooperation • lineage tracing	same as above	same as above	[85, 90–99]

Abbreviations ITH: Intra-tumoral heterogeneity, CSC: cancer stem cell, GEMM: genetically engineered mouse model, PDX: patient-derived xenograft

#### Genomic ITH in Primary Tumors

A landmark study of 21 primary breast tumors spanning the major breast cancer subtypes used whole-genome sequencing (WGS) to computationally model the evolutionary history of each tumor’s development. Each tumor analyzed harbored genomic ITH. In fact, the majority of somatic mutations detected were restricted to subclonal populations of tumor cells [16]. Further investigation of genomic ITH using WGS and targeted DNA multi-region sequencing of 50 primary breast tumors revealed that ITH was not evenly distributed amongst spatially distinct biopsies within the same tumor, suggesting that representative sampling of tumors is an important consideration when sampling tumors [15]. An expanded analysis of greater than 100 TNBCs revealed that while *TP53, PIK3CA*, and *PTEN* somatic alterations were recurrent amongst some patients and tended to be clonally dominant, a wide spectrum of ITH amongst patients was observed, with up to 19 genomic subclones observed in an individual’s breast tumor [17]. ITH of HER2 overexpression and gene amplification has been reported in HER2 + breast cancer patients. Whole exome sequencing (WES) and targeted sequencing of HER2 + breast cancers identified alterations in oncogenes including *BRF2* and *DSN1* in the HER2-negative compartment, while all samples were all ER-positive and predominantly harbored somatic *TP53* mutations [18].

A variety of computational algorithms have been developed to integrate this information to model subclonal architecture . Computational approaches have been used to reconstruct the subclonal history of tumors by deconvoluting DNA sequencing data from bulk tumors. Sequencing data reveal the mutant allele frequencies (MAFs), the fraction of reads containing mutant alleles, of the single nucleotide variants (SNVs), and the depth of the sequencing at a genomic location is associated with the local copy number. Using this data, methods have been developed to estimate subclonal architecture from SNVs (PyClone [100], BayClone [101], DPClust [16], Sciclone [102], cloneHD [103], CTPsingle [104], PhyloSub [105], PhyloWGS [106], LICHeE [107], BAMSE [108]), copy number profiles (Sclust [103], THetA [109], TITAN [110]), or structural variants (SVclone [111]).

By far, the most effort has been devoted to methods that rely on SNVs, likely due to the fact that SNVs are more directly observed in sequencing data than are CNAs or structural rearrangements. Nevertheless, constructing subclonal architecture has been challenging. Subclonal SNVs are estimated based on MAFs, which are proportional to the cancer cell fractions (*i.e. *the fraction of the cancer cells that comprise the subclone). More specifically, MAFs are a result of the purity of the tumor, the multiplicity of mutation (the fraction of the total copy number that is mutated), and the subclonal frequency. Unfortunately, the multiplicity of mutation is not observed, and each of the methods employ different assumptions and approaches to estimate this, leading to frequent disagreement in architectures. In practice, multiple methods are used to create a consensus architecture. While bulk sequencing approaches are readily available with existing technologies, the ability to detect rare subclone populations is limited. Indeed, the power to detect low-frequency mutations is highly dependent on depth of sequencing coverage [112]. Furthermore, clinically important samples, often from metastatic sites or therapy-resistant tumors longitudinally, are often not readily available. Thus, bulk sequencing is often considered a low-resolution approach suitable for detecting sizeable subclones or gross changes in subclonal composition across tumors.

#### Genomic ITH in Therapy Resistance

Genomic analyses of patients’ tumors under treatment have provided insights into the dynamics of ITH and identified genomic mechanisms of resistance. Although ITH is associated with poor prognosis and outcome in several tumor types [113–116], it is not yet clear whether higher degree of ITH in breast cancer is correlated with development of therapy resistance. Analysis of a relatively small sample set suggested the extent of genomic ITH of primary breast cancers was found to have no correlation with neoadjuvant chemotherapy response [15], while another study of a small cohort did suggest a correlation between the degree of pre-treatment ITH, as assessed by iFISH for several chromosomal probes, and chemotherapeutic responses [14]. A longitudinal study in metastatic breast cancer has shown that subclones harboring resistance pathways can become dominant after treatment with chemotherapy [34]. Thorough assessment of the relationship between ITH and therapeutic resistance will require genome-wide analyses of a greater number of biopsies associated with therapeutic response data. A study using targeted deep cancer gene sequencing of greater than 1900 breast cancers, most of which were hormone receptor positive, revealed enrichment of mutations in genes such as *ESR1* and *HER2* in post-endocrine therapy treated tumors relative to pre-treatment tumors. Additionally, alterations such as *EGFR* amplification or *HER2* mutation were detected in rare subpopulations of tumor cells prior to treatment and then became enriched following endocrine therapy in subsets of patients. In contrast, mutations in MAPK signaling genes such as *NF1*, *KRAS*, *MAP2K1*, and *BRAF* were typically not detected in the pre-treatment primary tumors, but rather arose *de novo* after therapy, suggesting some mutations were acquired while others were pre-existing then selected following endocrine treatment [19].

With the introduction of HER2-targeted therapies such as trastuzumab, overall survival has vastly improved in HER2 + patients [20]. However, multi-region WES pre- and post-neoadjuvant chemotherapy plus HER2-targeted treatment of five HER2 + breast cancer patients revealed extensive genomic ITH pre- and post-therapy. A subset of tumors exhibited shifts in clonal architecture and outgrowth of clones harboring *de novo* mutations, whereas others maintained stable clonal architecture, following treatment [21]. Therefore, despite the elimination of some therapy-responsive clones, resistant clones were able to survive and predominate the tumor cell population. Therapies aimed at targeting anti-HER therapy-resistant subclones will be necessary to overcome this ITH.

Chemotherapies are the only approved agents for treatment of non-BRCA1/2 mutant TNBC, and patients with substantial residual cancer burden following standard chemotherapy have poor relapse-free and overall survival rates [117]. Targeted cancer gene sequencing of > 80 post-chemotherapy residual tumors revealed that 90% harbored at least one clinically actionable genomic alteration, some of which were subclonal [22]. While ITH dynamics have been analyzed in only a limited number of pre- and post-chemotherapy TNBC biopsies, a few preliminary trends have emerged. First, targeted cancer gene sequencing of 20 matched pre- and post-chemotherapy biopsies revealed that while most cancer gene MAFs were unaltered following treatment, MAFs in genes including *ATM, TP53*, and *CDH1* became enriched after treatment in a fraction of patients [22]. Similarly, WES or WGS has been conducted on a combined total of 12 matched pre- and post-chemotherapy residual TNBCs, encompassing a variety of standard chemotherapies, revealing that while some patient’s tumors underwent clonal selection, others maintained similar genomic architecture after chemotherapy [22–24]. Larger studies investigating whether ITH dynamics are specific to chemotherapeutic agent, and whether they are functionally important, will provide valuable insights into mechanisms of chemoresistance in TNBC. In cases where genomic architecture is stable throughout treatment, non-genomic mechanisms of resistance such as metabolic and epigenetic plasticity have been identified as functionally contributing to survival of chemoresistant subpopulations in prelinical studies [24, 118].

#### Genomic ITH in Metastasis

Genomic studies of matched primary tumors and metastases have provided insights into the clonal origins of, patterns of seeding, and relatedness of distant metastases in breast cancers. The potential for a functional role of primary tumor ITH in the development of metastasis is not fully understood. One study utilizing iFISH-based genomic ITH estimation of 75 primary TNBC biopsies revealed that the extent of ITH in CNVs of MYC, EGFR, and CCND1 correlated with subsequent metastasis [33]. Although only a limited number of matched primary and metastatic tumors have been genomically analyzed, several informative findings have emerged. First, while metastases are clearly clonally descended from the primary tumor, harboring all clonal mutations found on the ‘trunk’ of the tumor’s evolutionary tree, they often harbor only subsets of subclonal mutations from primary tumors, indicating an evolutionary branch point [15, 25–28]. Second, most driver gene alterations are detected as evolutionarily early events in primary tumors and maintained in metastases, rather than arising *de novo* in metastatic lesions [15, 28]. Third, the majority of breast cancer metastases across subtypes are polyclonal, meaning they harbor genomic ITH [28], although this is a controversial topic across cancers and some evidence for monoclonal metastatic seeding has been found in breast cancer [29]. Fourth, a very limited number of studies have analyzed matched primary tumors and multi-site metastases within individual patients, enabling comparisons across secondary organs. WES and targeted sequencing of matched primary and multiple metastatic autopsy samples from 10 metastatic breast cancer patients revealed that metastasis-to-metastasis ‘secondary’ seeding may happen more frequently than multiple seeding events from the primary tumor [29]. With a set of 11,616 breast tumors, including 5,034 metastases, a recent study revealed heterogeneous status of *ER* and *HER2* mutations across metastatic sites. Some metastases were found to have distinct mutations, with enrichment of *ASXL1* amplification and *PTEN* deletion in brain, *DNMT3A* mutations in bone, *NOTCH1* mutations in skin, and *KRAS*, *KEAP1, STK11* and *EGFR* mutations in lung metastases [30]. These findings suggest that genomic alterations may enable breast tumor cells to adapt to distinct foreign microenvironments.

An important consideration in comparing matched primary and metastatic tumors is that patients often receive systemic treatments in the time between primary and metastatic biopsy sampling, thus the pattern of clonality in the metastatic site is likely influenced by these treatments. In fact, treated metastases have been found to be enriched for functional driver mutations compared to untreated metastases. Metastatic seeding can happen very early for synchronously diagnosed metastases while distant metastases following treatment occur relatively later and harbor more genomic aberrations including enrichment of functional driver mutations [31]. This implies that some treatments can remodel the clonal evolution of metastasis and may select disseminated cells harboring drug-resistant mutations. When combined with functional studies in laboratory models as reviewed below, genomic analysis of biopsies directly from patients can provide useful insights into the natural history of metastatic dissemination.

### Analysis of Liquid Biopsies From the Clinic

Due to spatial and temporal ITH, a single tumor biopsy may not fully represent the complete molecular profiles of all lesions within an individual patient [3]. While multiple biopsies are not always clinically, technically, or ethically feasible, longitudinal sampling of peripheral blood, known as “liquid biopsy”, is a minimally invasive approach to monitor tumor progression [119] and enables monitoring of genomic aberrations present in metastatic lesions [120]. Monitoring circulating tumor DNA (ctDNA) from early-stage breast cancer patients demonstrated sensitive detection of oncogenic mutations [121]. ctDNA analysis revealed within-patient ITH between the primary tumor, ctDNA, and metastases from metastatic breast cancer patients [49]. Serial analysis of ctDNA in an ER + breast cancer patient with synchronous bone and liver metastases captured all mutations from the primary tumor and liver metastasis and further revealed shifts in MAFs over time [50]. Monitoring clonal evolution by comparing plasma and tumor biopsies from a patient with metastatic ER+/HER+ breast cancer over three years identified serial changes in circulating levels of subclonal mutations, which correlated with differential targeted treatment responses between metastatic sites [51]. These studies demonstrated that ctDNA is a tool for longitudinal monitoring of multi-site disease progression throughout the course of targeted therapy treatments.

Recent studies have shown the feasibility of detecting *ESR1* mutations in ctDNA and provide a better understanding of the prevalence of *ESR1* mutations in various stages of disease and throughout treatment [52]. Comparison of the frequency of *ESR1* mutations among the primary tumor, multiple metastases, and in ctDNA revealed that mutation rates of *ESR1* in ctDNA are higher than in other lesions and lowest in the primary tumor [122]. ctDNA sequencing often identified multiple *ESR1* mutant alleles while only a single mutation was detected in each individual metastatic biopsy, suggesting that liquid biopsies may reflect *ESR1* mutational heterogeneity from diverse metastatic lesions [123]. A study of ER+/PR+/HER2 − metastatic breast cancer patients demonstrated the emergence of acquired *HER2* mutations in circulating tumor cells (CTCs) isolated from 143 patients who had developed endocrine therapy resistance. Treatment with neratinib, an irreversible HER2 inhibitor, resulted in significant clinical responses, supporting the use of CTC analyses to monitor molecular heterogeneity that emerges as tumors resist therapy [124].

## Bulk Analysis of ITH in Laboratory Models

While studies profiling biopsies from patients have extensively documented ITH in primary, therapy resistant, and metastatic breast cancer samples, laboratory models of breast cancer have provided further insights into the functional roles of heterogeneous tumor cell subpopulations. Experimental models enable tighter control of variables, an opportunity that is often not afforded in the clinical setting. Furthermore, use of biological replicates when monitoring ITH in experimental models enables determination of whether patterns of ITH, as well as how ITH responds to external pressures, are reproducible and thus potentially linked to functional biological features. In this section we review experimental approaches to study ITH using *in vitro* and *in vivo* models of breast cancer including cultured cell lines, GEMMs, and PDXs. While use of cell lines is ideal for ease of manipulation and testing of numerous experimental conditions, concerns over lack of microenvironmental factors and evolutionary selection of populations specifically suited to two-dimensional culture may limit the translatability of findings. GEMMs have an intact immune system and same-species interactions between tumor and stromal components. However, these models are generally initiated by a select few oncogenic driver events, progress over timescales much faster than do human tumors, and may not harbor the extent of complex ITH that is observed in human breast tumors. On the other hand, PDX models enable experimentation with highly heterogeneous populations of minimally manipulated human tumor cells. However, PDX models lack a fully intact immune system, have cross-species tumor-stroma cell interactions, and can inadvertently enrich for aggressive subclones from the originating human tumor. While each of these models has its limitations, they have each yielded valuable insights into breast tumor biology and ITH as discussed below (Table 1).

### Laboratory Models – Cancer Stem-Like Cell Hierarchies

According to the CSC model, ITH can be generated by subpopulation of TICs with self-renewal capabilities at the hierarchical apex of tumor cell differentiation. Numerous studies have documented the existence of CSCs using flow cytometry-based identification of cell surface markers. In PDX models representing the major breast cancer subtypes, cells expressing CD44 with low or no expression of CD24 (CD44 + CD24-) [4], as well as cells with aldehyde dehydrogenase (ALDH) activity [77], were found to be enriched for CSC features as evidenced by *in vitro* mammosphere formation capacity and *in vivo* tumor-initiating capacity. Gene expression profiling of breast cancer cell lines and PDX models revealed that CD44 + CD24- cells were enriched for expression of mesenchymal genes while ALDH-expressing cells had an epithelial gene expression profile [78]. Additionally, in ER/PR-negative xenografts, a population of TICs enriched in CD44 + CD49f^hi^CD133/2^hi^ cells displayed CSC features and was characterized by heightened expression of the stem cell-associated genes *BMI1*, *NANOG*, and *SOX2* [79].

In a panel of GEMMs of p53-null transplantable mammary tumors, CSC features were enriched in the lineage (Lin)^−^CD29^Hi^CD24^Hi^ subpopulation which displayed heightened resistance to radiotherapy and activation of the Akt pathway. Treatment with an Akt inhibitor sensitized TICs to radiotherapy, suggesting targeting CSCs may be of potential therapeutic benefit [59, 60]. In the mouse mammary tumor virus (MMTV)-Wnt1 GEMM, the CD24 + Thy1 + subpopulation is enriched for CSC properties [61]. Several additional markers have been shown to enrich for the CSC population in the MDA-MB-231 cell line model of TNBC, including CX26 [90], LGR5 [91], ANTXR1 [92], OCT4A [93], HN1L [94], RPL39, and MLF2 [95]. Together, these studies have demonstrated that ITH of TIC and/or CSC features is prevalent in breast cancers and that therapeutic targeting of the CSC subpopulation may be a promising approach to eliminate the self-renewal capacity of breast tumors.

### Laboratory Models – Inter-clonal Interactions

A consequence of ITH, potentially amplifying its malignancy, is the communication and cooperativity between distinct cell subpopulations. In the MMTV-Wnt1 mouse mammary tumor model, mammary-specific expression of the *Wnt1* oncogene produces tumors with a mixture of luminal and basal epithelial cell populations. Transplantation of fluorescence-activated cell sorting (FACS)-isolated populations (Wnt1-secreting luminal cells or basal cells that had spontaneously acquired an *Hras* mutation), into mammary fat pads revealed that animals receiving either subpopulation alone failed to develop tumors, whereas transplantation of an admixture of the two subpopulations led to tumorigenesis [62]. Similarly, generation of secreted factor-overexpressing subclones from the MDA-MB-468 human TNBC cell line followed by mammary fat pad transplantations revealed non-cell autonomous maintenance of ITH and promotion of tumor growth through interleukin 11 stimulation of tumor-promoting changes in the local vasculature [125]. Isolation of the (Lin^−^)CD29^hi^CD24^hi^ population from p53-null mouse mammary tumors by FACS followed by gene expression profiling revealed mesenchymal gene expression patterns characterized by Wnt and cytokine signaling [59]. These mouse models enabled functional interrogation of mesenchymal stem cell niche cells and TICs. FACS isolation of each population from mammary tumors followed by single or co-culture of each population revealed that the proliferative and self-renewal properties of TICs were enhanced when co-cultured with niche cells or niche cell-conditioned medium. These properties were also observed when orthotopically transplanting *in vivo*. Lentivirus-mediated knockdown of *Wnt2* within the niche population led to reduced self-renewal capabilities of the TIC population *in vitro* and *in vivo* [63]. Thus, generation of these mouse models harboring ITH enabled functional dissection of the role of various tumor cell subpopulations. *In vitro* assays, using experimentally produced trackable clones derived from the MDA-MB-231 cell line revealed interactions between clones functionally drove tumor growth, implying inter-clonal communication may play a functional role in tumor progression [85]. Thus, studies in a variety of experimental models have demonstrated the pro-tumor functional effects of ITH.

### Laboratory Models – Genomic ITH

Genomic sequencing has enabled computational modeling of subclonal architecture in PDX models, revealing extensive genomic ITH and distinct patterns of clonal dynamics across samples [25, 70–72]. Analysis of genomic ITH in serially passaged PDXs revealed that while some models maintained stable levels of minor subclones relative to matched patient tumors, other models exhibited a selective sweep in which a minor subclone outgrew at early or late passages [71]. Thus, while some PDX models faithfully maintain the genomic ITH of patients’ biopsies, others do not. Mouse models of spontaneously arising metastasis have enabled investigation of the somatic evolution of breast tumor cells throughout the metastatic cascade. Next-generation sequencing of two luminal-like breast cancer GEMMs, MMTV-PyMT and MMTV-Her2, revealed a high degree of genomic ITH within each model. Analysis of the functional relevance of CNAs identified two potential metastasis-related genes, *Col1a1* and *Chad*. A population of cells with *Col1a1* and *Chad* knockdown were unable to metastasize to the lung after orthotopic injection, suggesting these are functional metastasis driver genes [56]. A study that conducted WES of paired primary tumors and lung metastases from the MMTV-PyMT and MMTV*-*Her2 models identified metastasis-enriched and metastasis-specific mutations and CNAs in known oncogenes, including *Kras.* Orthotopic engraftment of cells expressing the *Kras* G12D mutation led to increased metastatic burden while knock down of *Kras* reduced metastasis to the lungs [126].

Many orthotopic PDX models develop spontaneously arising metastases to common organ sites of human breast cancer metastasis [73, 127–129]. Genomic analyses of matched primary tumors and metastases from PDX models have revealed that despite the high degree of genomic similarity, metastases often arise from a minority of primary tumor subclones as evidenced by differential maintenance of MAFs and CNAs. These analyses of PDX models have also revealed that the majority of mutations detected in metastases were also detected, albeit at lower frequencies, in the matched primary tumor [25, 73, 130]. Together, these studies have revealed that metastases are clonally derived from primary tumors, often from a minor primary tumor subclone, and that metastatic cells sometimes continue to evolve *de novo* genomic aberrations after escaping the primary tumor. Deep sequencing of multi-site metastases in a PDX model of TNBC revealed reproducible selection of a common genomic subclone in lung, liver, and brain metastases relative to primary tumors, suggesting that distinct organ microenvironments may enable outgrowth of shared genomic subclones [73]. Isolation of CTCs from TNBC PDX models revealed CTC clusters harbored heterogeneous levels of epithelial and mesenchymal proteins, suggesting that CTCs in PDX models harbor phenotypic ITH [74]. To address the functional role of ITH in metastasis, the orthotopically xenografted MDA-MB-468 cell line model, in which secreted factor-over-expressing subclones were derived, was used to demonstrate that polyclonality was required for metastatic outgrowth. This study revealed that two factors, IL11 and FIGF, secreted by minor primary tumor subclones, promoted distant metastases through effects on the immune and vascular microenvironments, respectively [131].

Studies investigating CSC features and genomic ITH have provided insights into mechanisms of therapy resistance. Based on the observation that TNBC cells express high levels of EGFR protein and reactive oxygen species, treatment of MDA-MB-468 TNBC cells with an antioxidant in combination with an EGFR inhibitor revealed a marked growth inhibitory response. Mammosphere-forming cultures of FACS-sorted CSC and non-CSC cells revealed that the EGFR inhibitor targeted non-CSC cells while the antioxidant targeted the CSC sub-population, suggesting that combining drugs to target heterogeneous tumor cell subpopulations may be an effective therapeutic strategy [84]. Genomic sequencing of PDX tumors throughout treatment with standard chemotherapies revealed that TNBCs can maintain genomic ITH rather than exhibiting a selective bottleneck or evolution of *de novo* genomic aberrations [24]. This non-mutational mode of resistance has been described as a reversible drug-tolerant state and can be mediated by alterations in tumor cell metabolism [24] and epigenetic programs [132]. In summary, analyses of genomic ITH in laboratory models have demonstrated reproducible patterns of subclonal architecture and dynamics with a high degree of similarity to those observed directly in patients’ biopsies and allow for functional dissection of heterogeneous subpopulations.

### Laboratory Models – Lineage Tracing

Lineage tracing approaches have enabled precise monitoring of ITH dynamics in a variety of *in vitro* and *in vivo* experimental models. One, or up to thousands of, tumor cell subpopulation(s) can be labeled with a tag transmitted to all cells clonally descended from the initially tagged cell which is then detected by fluorescence imaging or sequencing. These approaches have effectively provided quantitative portraits of subclonal architecture in multiple laboratory models of breast cancer.

#### Multi-color Lineage Tracing

GEMMs have enabled fluorescence *in situ* lineage tracing of luminal and basal cell lineages throughout tumorigenesis, revealing that the identity of the cell of origin bearing a tumor-imitating oncogenic insult can dictate mechanisms of tumorigenesis and progression [64]. Intravital imaging enabled monitoring of cancer stem-like cell plasticity longitudinally without the need to disrupt and isolate tumor cell subpopulations and revealed that TIC properties could be dynamically lost and gained within diverse subpopulations of cells over the course of mammary tumor growth. Using the MMTV-PyMT model [65], investigators introduced a previously generated Cre-inducible ‘confetti’ construct [133] which randomly induces expression of CFP, GFP, YFP, or RFP in each transduced cell. In this system, clonal expansion of a founder TIC manifests as large tumor areas marked by a single fluorophore. Longitudinal intravital imaging provided experimental evidence for the plasticity of TIC capacity, as evidenced by loss of some single-color areas and gain of *de novo* single-color areas over several weeks of mammary carcinoma development [134]. Imaging of single cells in the complex 3D environment of intact breast tumor tissue has been a major challenge, but recent advances in tissue clarifying methods that preserve tissue and cellular architecture have provided the unprecedented ability to image ITH in tissues. This method enabled multiplexed 3D immuno-fluorescence imaging of a PDX breast tumor, revealing extensive cellular and spatial ITH. Authors also applied this methodology to GEMMs bearing the Cre-inducible ‘confetti’ construct [133] in luminal or basal lineages to visualize 3D ITH throughout tumorigenesis [66]. Further utilization of multi-color lineage tracing constructs with the advancement of 3D single-cell imaging will provide invaluable insights into ITH dynamics following therapeutic treatments and throughout the metastatic cascade.

While numerous genomics studies have demonstrated that metastases are usually polyclonal, multi-color lineage tracing in the MMTV-PyMT mouse model allowed direct testing of the relative metastatic capacity of single vs. clustered tumor cells [67]. This study used a two-color inducible construct that switched from membrane tdTomato to membrane eGFP expression upon introduction of adenoviral Cre recombinase, thus allowing determination of whether tumor cells detected throughout the metastatic cascade were single or two-colored. This approach identified multiclonal tumor cell clusters at various stages of metastasis and revealed that clusters were substantially more metastatic than were single tumor cells. This system has also been used in PDX models of TNBC to track the fates of cells positive for epithelial-mesenchymal transition (EMT)-related gene expression, revealing that only subsets of EMT-related genes were associated with *in vivo* tumor-initiation capacity [80]. A recent study reported the utility of immunodeficient zebrafish engrafted with a wide array of human cancer cells, including breast, which exhibited similar growth kinetics to cells grown in immune-compromised mice. Zebrafish are optically clear and thus permit ready longitudinal fluorescence imaging with single-cell resolution. This study demonstrated the power of this system to monitor ITH dynamics following therapeutic treatment of zebrafish engrafted with rhabdomyosarcoma cells that had been engineered to express a four-color cell cycle reporter [81]. Implementation of this model system to study ITH of breast cancer cells undergoing clinically relevant therapeutic regimens will be of great interest.

#### Viral Barcode-mediated Lineage Tracking

Several studies have leveraged lentivirally introduced lineage tags, or “barcodes”, to simultaneously track the dynamics of hundreds to thousands of clonal lineages in breast cancer models. Barcode tags are neutral, short DNA sequences that virally integrate into random genomic locations. Pooled barcode libraries enable high-throughput tracing of clonal lineages when transduced at a low multiplicity of infection such that the vast majority of transduced cells receive one single barcode, followed by next-generation sequencing to identify and quantify barcodes [135]. While barcodes enable high-throughput analysis of hundreds to thousands of unique clones simultaneously, they are not directly linked to molecular events within the cells they mark, necessitating further downstream investigations to identify drivers of the observed patterns of ITH. This technology has been used in cell line and PDX *in vivo* models of breast cancer to track patterns of ITH during xenograft passaging and primary tumor growth, revealing substantial ITH and diversity of clonal growth patterns between samples derived from distinct models [82]. Retroviral barcoding of a breast cancer cell line *in vitro* revealed that uniquely barcoded subclones maintained diverse ratios of epithelial and mesenchymal subpopulations, revealing phenotypic plasticity within each clonal lineage [96]. As an alternative approach to random integration of lentiviral barcode libraries, the CRISPR/Cas9 system has been used to introduce genomic scars, serving as unique lineage barcodes, at a defined genomic location based on small guide RNA recognition. Introduction of a complex library of CRISPR barcodes in the BT474 breast cancer cell line revealed distinct maintenance of barcoded subpopulations when cells were grown *in vitro* or orthotopically xenografted, suggesting subpopulations of clones harbored an intrinsically distinct *in vivo* growth potential [97].

Barcoding has been used to quantitatively monitor development of therapy resistance in models of breast cancer. High-complexity *in vivo* barcode tracking in orthotopic PDX models of primary untreated TNBC revealed maintenance of barcode complexity, rather than a selective bottleneck, following treatment of mice with standard front-line chemotherapies. Rather than genomic evolution, resistance in these models was found to be mediated by a phenotypic state characterized by a metabolic and epigenetic adaptations [24]. High-complexity barcoding of the ER + MCF7 cell line treated with fulvestrant or tamoxifen *in vitro* revealed selection for a pre-existing resistant subclone, whereas treatment with an inhibitor of the KDM5 histone demethylase resulted in no clonal selection [98]. Together, these data indicate that patterns of clonal dynamics are likely specific to the breast cancer subtype and mechanism of action of therapy.

Barcoding approaches have provided valuable insights into the natural evolution of primary breast tumor cells as they metastasize in human and mouse tumor models. *In vivo* barcoding with a highly complex library in orthotopic PDX models of primary untreated TNBC revealed a selective bottleneck when comparing lung, liver, and brain metastases to primary tumors. The natural occurrence of multi-site metastases within these models enabled direct comparison of spatially distinct metastases, revealing enrichment of shared clonal lineages across diverse secondary organ microenvironments. The high complexity of the barcode library enabled detection of extremely rare populations of seeding clones that were maintained at low levels in metastatic sites, further providing evidence for polyclonal seeding [73]. Similarly, *in vivo* barcoding of PDX models with a lower-complexity library revealed that while chemotherapy had little impact on ITH, metastasis imparted a selective bottleneck [83]. Retroviral introduction of barcodes into the mouse mammary tumor-derived 4T1 cell line followed by orthotopic engraftment into immune-compromised mice enabled comparison of lymphatic metastases, CTCs, and blood-borne metastases in the lung, liver, and brain. Of primary tumor clones, only a subset was detected in CTCs, a subset of which was detected in blood-borne metastases. These lineages were largely non-overlapping with those found in lymph node metastases [99]. Together, these studies have demonstrated strong shifts in the spectrum of ITH in multi-site metastases of diverse breast cancer models. Future clonal tracking studies using syngeneic mouse models and xenograft models with humanized immune systems will enable evaluation of the role of immune cell populations in the subclonal dynamics of metastasis.

#### Developmental Barcoding

Developmental barcoding strategies using engineered CRISPR/Cas9 systems have been devised to monitor embryonic development in a variety of model systems [136–140]. These models will likely yield profound insights into tumor subpopulation dynamics when crossed with mouse mammary tumor models. One such study conducted lentiviral injection of the amniotic cavity of mouse embryos to introduce barcodes into mammary epithelial progenitor cells, which were found to have equal likelihood to give rise to myoepithelial or luminal cells in adult mammary ducts. This barcoding strategy was then used to screen cancer genes by introducing a barcoded pooled lentiviral library of shRNAs and ORFs against putative cancer-related genes into the Keratin 14-Cre; Pik3ca^H1047R^ tumor model following amniotic injection. This approach identified several genes whose manipulation accelerated tumor formation [68]. Thus, this novel method enables both neutral barcode-mediated lineage tracing and high-throughput *in vivo* screening in an immune-competent mouse background. Application of this methodology to additional breast cancer GEMMs that enable analysis of therapy-resistant and metastatic tumors will likely provide valuable insights into clinically relevant drivers of ITH in breast cancer.

## Single-cell Methods to Assess ITH

### Single-cell Genomics and Transcriptomics of Tumor Biopsies From the Clinic

The development of single-cell technologies has vastly improved our ability to detect and characterize ITH. Single-cell sequencing overcomes many of the major limitations faced when sequencing bulk samples, namely that individual subclones no longer need to be deconvoluted computationally. A number of single-cell sequencing technologies have been developed that can assay a range of molecules, including DNA [141], RNA [142], methylation [143], or chromatin accessibility [144]. The main limitation of these approaches is the cost, which restricts the number of cells that can be profiled, and thus, the sizes of the cell populations that can be found. To date, most single-cell sequencing studies have characterized primary tumor samples. Some of the earliest studies using single-cell DNA sequencing approaches in ER + and TNBC specimens and a paired liver metastasis, clearly confirmed the presence of extensive genomic ITH in each sample. These studies provided early evidence that tumors may evolve early in punctuated bursts of CNAs followed by gradual accumulation of mutations [35, 36]. Single-cell genomic sequencing has also enabled characterization of disseminated breast tumor cells isolated from bone marrow [37]. A recent study using single-cell genome sequencing of primary tumors collected from 16 patients revealed pervasive ITH of CNAs, often differing between two spatially resolved biopsies, highlighting the importance of performing spatial sampling [38]. Spatially resolved single-cell DNA sequencing, enabled by microdissection of formalin-fixed paraffin embedded (FFPE) samples of ductal carcinoma *in situ* (DCIS), revealed genomic ITH throughout DCIS progression [39]. Furthermore, single cell sequencing confirmed that a shared genomic lineage led from the DCIS to invasive lesions and that the majority of genomic aberrations occurred in DCIS prior to acquisition of invasive properties [40]. In recent years, single-cell RNA sequencing revealed heterogeneity of transcriptomic profiles amongst tumor and stromal cells [41, 42]. Laser capture microdissection was used to isolate tumor and stroma regions in tissues from TNBC patients. Microarray gene expression analysis of microdissected regions revealed unique profiles associated with distinct tumor immune-microenvironment spatial arrangements. Infiltration of CD8 + T cells within the tumor mass, rather than being restricted to the tumor periphery, was found to be correlated with good outcome. In contrast, an “immune-cold” microenvironment was associated with poor outcome [43]. In addition, single-cell RNA sequencing of tumors collected from 5 TNBC patients enabled resolution of tumor and stromal subpopulations, revealing extensive ITH of cancer-associated fibroblasts (CAFs) and their expression of specific transcription factors [44].

Single-cell analysis has also provided new insights into whether drug resistance is caused by the selection of pre-existing resistant clones or through acquired resistance following treatment. Findings from iFISH with several probes against frequently amplified chromosomal regions of pre- and post-NACT tumor biopsies from 47 breast cancer patients (13 luminal A, 11 luminal B, 11 HER2+, and 12 TNBCs) showed that tumors with complete response had low pre-treatment genetic diversity of chromosomal probes, whereas tumors with higher genetic diversity only had partial responses to chemotherapy. Importantly, patterns of genetic diversity were unchanged pre- and post-NACT in tumors with partial responses. However, patterns of ITH based on expression of CSC markers CD44 and CD24 were altered following NACT, suggesting that cellular phenotypes are adaptable in the absence of genomic evolution [14]. A study using specific-to-allele PCR-FISH (STAR-FISH) from treatment-naïve HER2 + samples revealed that a minor subpopulation of cells with a preexisting *PIK3CA* mutation were enriched following chemotherapy. Furthermore, the spatial distribution of *PIK3CA* mutant cells and HER2-amplified cells was predictive of trastuzumab responses [45]. Single-cell DNA sequencing of longitudinal samples from TNBC patients before and after chemotherapy revealed that in some patients, resistant genotypes pre-existed in tumors prior to treatment. Interestingly, single-cell RNA-sequencing in a subset of the same samples showed that the transcriptome profiles of cells pre-treatment and post-treatment were distinct and that transcriptomic programs found in post-chemotherapy residual tumors were undetectable before treatment [23, 34]. Together, these studies suggest that chemoresistance is sometimes associated with both genomic selection and transcriptomic reprogramming.

Studies of RNA expression profiles in individual CTCs have demonstrated the existence of transcriptomic ITH in liquid biopsies [53]. Single-cell RNA-sequencing identified that rare CTC clusters harbored unique gene expression programs and were more likely to initiate metastasis compared to single CTCs. A CTC-cluster-enriched gene, plakoglobin, was expressed heterogeneously in primary and metastatic breast tumors, suggesting its expression may be restricted to the subpopulation of primary tumor cells harboring metastatic capacity [54]. Profiling CTCs from patients with metastatic breast cancer revealed development of mutations and alterations within *ESR1* in individual CTCs during endocrine therapy, suggesting mechanisms of endocrine therapy resistance [55]. Together, these studies have demonstrated that single-cell analyses enable precise delineation of genotypes and phenotypes associated with metastasis and therapy resistance.

### Single-cell Proteomic Measures of Tumor Biopsies From the Clinic

While mass spectrometry proteomics provide high-resolution analysis of protein levels in bulk samples, these methods are not yet fully developed for single-cell analyses to appreciate ITH. Rather, several approaches have been developed to analyze levels of select proteins using antibody-based detection on tissues or in cell suspensions. Newly developed mass cytometry approaches, in which cell suspensions or tissues are stained with heavy metal isotope-conjugated antibodies against up to ~ 40 different proteins simultaneously, have enabled detection of complex proteomic ITH in breast cancer samples. A study using cell suspension cytometry time of flight (CyTOF) with tumor and stromal cell-specific antibody panels revealed extensive proteomic and cell type heterogeneity in cell suspensions obtained from 144 tumors of breast cancer patients and 50 non-tumor tissues. Specifically, high frequencies of PDL1+ tumor-associated macrophages and exhausted T cells were found in high-grade ER + and ER- tumors, highlighting tumor-stroma interactions associated with immunosuppression and poor prognosis [46]. Spatially resolved mass cytometry on tissue sections with a panel of 35 biomarkers using imaging mass cytometry (IMC) of 281 patients revealed 18 novel subgroups of breast cancer associated with distinct clinical outcomes [47]. By coupling IMC with a panel of 37 biomarkers and genomic profiles from the METABRIC cohort [12], a recent study revealed associations between genomic alterations and proteomic features of tumor ecosystems, including cellular interactions and neighborhoods linked to prognosis. In particular, mutations in *TP53* were associated with distinct epithelial and fibroblast phenotypes, suggesting that somatic genomic aberrations exert influence over the cellular compositions of both tumor and microenvironmental populations. Furthermore, epithelial cells that expressed a marker of hypoxia were associated with copy number gains of *CD274*, encoding PD-L1, and heterozygous deletions of *B2M*, encoding β2-microglobulin, suggesting that a hypoxic microenvironment may select genomic alterations that produce immune-tolerance [48]. Thus, ecosystem-based patient classification may facilitate identification of individuals for precision medicine approaches targeting the tumor and its unique microenvironment. While these approaches are providing unprecedented insights into proteomic and spatial complexity of breast tumors, current technologies are able to simultaneously assay a limited number of molecules, necessitating the *a priori* decision to assay pre-defined pathways or proteins.

### Single-cell Approaches in Laboratory Models

Studies focusing on cultured breast cancer cells have identified extensive genomic ITH within cell lines [86, 87]. Identification of ITH among clones from MDA-MB-231 TNBC cells revealed that differences in gene expression among distinct clones impacted cytokine- signaling between cells [85]. Single-cell sequencing of human breast cancer cell lines has enabled testing of the role of non-genetic ITH in therapeutic resistance. Single-cell RNA-sequencing revealed a heterogeneous response to glucocorticoids in a breast cancer cell line (T47D). Steroid hormone receptors such as the glucocorticoid receptor (GR) mediate transcriptional responses to hormones and are frequently targeted therapeutically. However, due to cell-to-cell variability in hormone responses, individual hormone-treated cells expressed only up to 30% of the total set of GR target genes. Understanding the basis of this heterogeneity will be critical for the development of more precise models of targeting steroid hormone signaling [88]. Single-cell RNA sequencing of the ER + MCF7 cell line revealed transcriptional variability within a rare subpopulation of therapy pre-adapted cells which underwent further transcriptomic reprogramming and CNAs to acquire full resistance to endocrine therapy, emphasizing the necessity of stage-specific biomarkers for studying multi-step models of therapy resistance development [89].

Single-cell methodologies are beginning to be leveraged in mouse models as well. Single-cell RNA-sequencing of Neu, PyMT, and BRCA1-null mouse mammary tumor models revealed that various tumors contained distinct CSC-like subpopulations driven by different oncogenic pathways [57]. Single-cell RNA-sequencing of the murine M6 allograft model of low-grade TNBC demonstrated that smoothened inhibitors reversed hedgehog pathway gene expression in CAFs that were responsive to the Hh ligand. Activated CAFs in turn provided a supportive niche for neoplastic cells to acquire a chemoresistant CSC phenotype, suggesting a therapeutic benefit from targeting of CAFs [58]. Recently, a new spatial approach to isolate and characterize tumor-proximal stromal cells from the metastatic niche has been demonstrated using diffusible fluorescence labeling of metastatic tumor cells [69]. Use of PDX models enables ready acquisition of *bona fide* human tumor cells in sufficient quantities for single cell analyses. Single cell DNA sequencing of PDX models revealed extensive genomic ITH, confirming observations made by bulk DNA sequencing studies. Single-cell targeted gene expression profiling in FACS-sorted PDX cells from early-stage (low burden) metastatic tissues, including lung, lymph node and brain, showed increased expression of genes involved in CSCs [71] and EMT while advanced lung metastasis cells were similar to primary tumors in their gene expression profiles [75]. Single-cell RNA-sequencing of matched primary tumors and lung micro-metastases from TNBC PDX models revealed transcriptomic ITH in primary tumors and lung micro-metastases. Metastatic tumor cell subpopulations harbored distinct levels of activation of gene expression pathways such as oxidative phosphorylation, highlighting the importance of monitoring ITH of gene expression programs to combat metastasis [76].

The recent development of single-cell analyses to identify epigenetic ITH has provided previously unachievable insights into non-genomic mechanisms of therapy resistance. Single cell methods to analyze epigenetic landscapes, such as single cell methylation sequencing, are beginning to be leveraged in other tumor types [145]. These methods have not yet been broadly applied to breast cancer. One study utilizing single cell combinatorial indexing Assay for Transposase Accessible Chromatin sequencing (sciATAC-seq) in PI3K/mTOR inhibitor-, and MEK inhibitor-treated TNBC cell lines to assess chromatin accessibility in individual cells revealed that distinct cell-state transitions arose in the absence of Darwinian selection of pre-existing subpopulations. Co-treatment with a PI3K/mTOR inhibitor and the BET inhibitor prevented the acquisition of a drug-tolerant persister state, resulting in cell death *in vitro* and xenograft regression *in vivo* [118]. A study using single-cell RNA-sequencing, cellular barcoding, and mathematical modeling demonstrated that endocrine resistance was due to pre-existing genetically distinct cells or acquired from alterations in activity of KDM5A/B, a histone demethylase involved in transcriptional regulation and DNA repair. In addition to genetic deletion of KDM5A/B, inhibition of KDM5 activity increased sensitivity to anti-estrogens by modulating ER signaling [146]. Collectively, these findings highlight the importance of understanding phenotypic and epigenetic heterogeneity in therapeutic resistance. Broadened use of single-cell epigenetic analysis methods is likely to provide valuable insights into mechanisms of therapeutic resistance.

## Concluding Remarks

Our understanding of ITH in breast cancer has progressed dramatically in the past decade and is likely to evolve further with technological advances and the development of novel experimental models. One of the most critical remaining questions is the correlation of ITH with therapeutic response and metastasis. While a few studies have preliminarily addressed this strictly at the level of analyzing genomic ITH, this needs to be addressed in larger cohorts and with methodologies revealing transcriptional, epigenetic, and proteomic ITH. These findings could provide novel methods with which to stratify patients, predict outcomes, and personalize therapies. However, the practical application of many approaches discussed herein, such as single-cell sequencing, to clinical decision-making is currently limited due to cost, throughput, sample availability, and limit of detection for low-abundance subpopulations (Table 1).

Numerous studies have demonstrated that tumors from laboratory models exhibit patterns of ITH that are similar to those seen in clinical biopsies. While clinical biopsies will always the gold standard against which findings from laboratory models should be compared, models enable functional studies and use of biological replicates that are impossible in the clinical setting. While each model has drawbacks, each has also proven useful for monitoring various aspects of ITH and tumor progression. The major benefits of *in vitro* models are the ease of manipulation for large-scale experiments, low time requirements, and low cost; these benefits come at the expense of a stromal microenvironment and the complete repertoire of ITH characteristic of human tumors. GEMMs offer the major benefit of harboring an intact immune microenvironment. These models, despite the fact they are mouse and not human tumors, have captured some aspects of ITH at the level of DNA aberrations, transcriptomic programs, and CSC phenotypes. However, these models are usually initiated in a relatively short time scale by one or a few oncogenic insults, thus narrowing the degree of ITH that can be observed. PDX models can capture a high degree of complex ITH of the patient tumor from which they were derived and thus are an effective model with which to experimentally monitor and perturb ITH in human tumor cells. As the major limitation of PDX models is lack of an intact immune system, use of mice with ‘humanized’ immune systems [147] will help to overcome this limitation, although these models are not yet amenable to large-scale studies. Furthermore, utilizing *in vitro* patient-derived organoids to increase throughput [148] enables experimentation on *bona fide* human tumor cells while sparing the need for large animal cohorts.

The majority of ITH studies using bulk and single-cell technologies have focused on genomic ITH. These studies have provided quantitative portraits of breast ITH and its dynamics throughout tumor progression. A major area of future work will entail expanded analyses of matched primary and multi-site metastatic lesions, as well as therapy-naïve and therapy-resistant residual tumors, to build upon the limited number of matched sample sets that have been analyzed thus far. These studies have the potential to elucidate patterns of tumor evolution that may be specific to or shared between distinct secondary organs, as well as patterns that may arise specifically in residual cells surviving therapies, and thus may reveal therapeutic targeting opportunities.

Fewer ITH studies have focused on transcriptomic, epigenetic, metabolic, or proteomic ITH, despite the fact these elements are likely to shed light on targetable cellular phenotypes. The full scale of proteomic and spatial ITH in breast cancer is only beginning to be appreciated, especially in the context of therapy-resistant and metastatic tumors. Several recent studies have addressed the possible influence of metabolic ITH in breast tumor biology [24, 149, 150]. The development of single cell metabolomic technologies is expected to provide novel insights into mechanisms driving breast cancer progression. Further investigation of molecular ITH with spatial resolution, such as with IMC, should provide valuable insights into the phenotypic evolution of resistance and metastasis in breast cancer. It will be vitally important that studies of these are expanded to include antibody panels testing a variety of cancer-related pathways relevant to therapy resistance and metastasis.

To identify approaches to therapeutically target heterogeneous tumors, new methods are needed. For instance, combinatorial CRISPR-based screens may identify effective means with which to target ITH in breast tumor samples [151]. Large-scale drug screening platforms, such as those enabling high-throughput two-drug combination testing in PDX-derived cells [152], hold promise to identify combinations that are synergistically cytotoxic across multiple models. Use of cells that can be subsequently xenografted allows for *in vivo* validation of *in vitro* drug screen hits, further increasing the probability of clinical translation. Thus, combining knowledge of clinically relevant signatures of ITH with multiplexed genetic and pharmacologic screening platforms holds great promise to combat therapy resistance and metastasis in breast cancer.
